# Complete Esophageal Obstruction Due to Dysphagia Lusoria and Subclavian Artery Thrombosis: A Case Report

**DOI:** 10.7759/cureus.78840

**Published:** 2025-02-11

**Authors:** Rim A Boutari, Moustafa W Diab, Fatmeh I Mallah

**Affiliations:** 1 Gastroenterology and Hepatology, Al Zahraa University Medical Center, Beirut, LBN; 2 Radiology, Lebanese University Faculty of Medicine, Beirut, LBN

**Keywords:** aberrant right subclavian artery, arsa, dysphagia lusoria, esophageal obstruction, thrombosis, vascular anomaly

## Abstract

Dysphagia lusoria is a rare condition caused by esophageal compression from an aberrant right subclavian artery (ARSA). Although often asymptomatic, complications such as aneurysmal dilation or thrombosis can result in severe presentations, including complete esophageal obstruction.

Herein, we report the case of a 75-year-old woman with a five-year history of progressive dysphagia and significant weight loss. During her hospitalization for urosepsis and acute kidney injury, she developed complete esophageal obstruction, preventing oral intake. Imaging revealed an ARSA with significant parietal thrombosis, compressing the esophagus posteriorly. This confirmed the diagnosis of dysphagia lusoria complicated by ARSA thrombosis. Conservative measures failed, and she was referred for vascular surgical evaluation.

This case highlights a rare but severe presentation of dysphagia lusoria with complete esophageal obstruction due to ARSA thrombosis. Early recognition and multidisciplinary management are critical for optimizing outcomes. Definitive treatment often requires surgical intervention, particularly in the setting of complications like thrombosis.

## Introduction

Dysphagia lusoria is a rare condition characterized by difficulty swallowing due to esophageal compression by an aberrant right subclavian artery (ARSA) [1]. This vascular anomaly, resulting from embryologic development variations, is present in approximately 0.5% to 1% of the population [2].

Interestingly, the subclavian artery on the left side derives from the left seventh intersegmental artery, while the right subclavian artery originates from the right fourth aortic arch artery and the right seventh intersegmental artery. Variations in this embryologic development can lead to an aberrant subclavian artery, which may compress the esophagus or trachea, causing dysphagia lusoria [3].

Dysphagia lusoria can be classified into different types based on the course of the aberrant artery, including retroesophageal and retrotracheal types. The presence of Kommerell's diverticulum, a dilation at the origin of the aberrant artery, can further complicate the condition and contribute to the symptoms [4].

This anatomical variant can be associated with other vascular malformations, such as tetralogy of Fallot, and may be present in syndromes including Turner, Edwards, Down’s, Di George, Patau, Potter, Noonan, and post-rubella syndromes [5].

Although many individuals with an ARSA remain asymptomatic, the onset of dysphagia, particularly in older adults, can be attributed to age-related changes such as decreased esophageal motility or arteriosclerotic alterations in the aberrant vessel [5].

In this report, we present the case of a 75-year-old female patient who experienced progressive dysphagia. Subsequent imaging studies revealed an ARSA causing significant esophageal compression, consistent with dysphagia lusoria. This case underscores the importance of considering vascular anomalies in the differential diagnosis of dysphagia, especially when routine evaluations do not reveal an obvious cause.

Early recognition of vascular anomalies, such as an ARSA, through appropriate imaging plays a crucial role in the evaluation of dysphagia, particularly when routine investigations do not reveal an obvious cause [6].

## Case presentation

A 75-year-old woman with a medical history of hypertension, coronary artery disease, and diabetes mellitus was admitted for urosepsis, septic shock, and acute kidney injury. During hospitalization, she reported a five-year history of progressive dysphagia to solids and liquids, accompanied by an unintended weight loss of approximately 15 kilograms. Despite the chronic nature of her symptoms, she had not sought prior medical evaluation.

On examination, the patient appeared cachectic and malnourished, with signs of dehydration. No neck masses or lymphadenopathy were noted. Laboratory studies revealed leukocytosis consistent with sepsis and hypoalbuminemia, reflecting her nutritional status (Table 1).

**Table 1:** Comparison of patient's laboratory findings with normal reference ranges CRP, C-reactive protein; WBC, white blood cell count

Lab test	Patient’s value	Normal range
WBC (×10^3^/µL)	16.2	4.0-11.0
Neutrophils (%)	86	40-75
CRP (mg/L)	65	< 5
Albumin (g/dL)	2.7	3.5-5.0
Total protein (g/dL)	5.0	6.0-8.3
Hemoglobin (g/dL)	9.0	12-16 (females) / 13-17 (males)
Creatinine (mg/dL)	1.53	0.6-1.2

**Figure 1:**
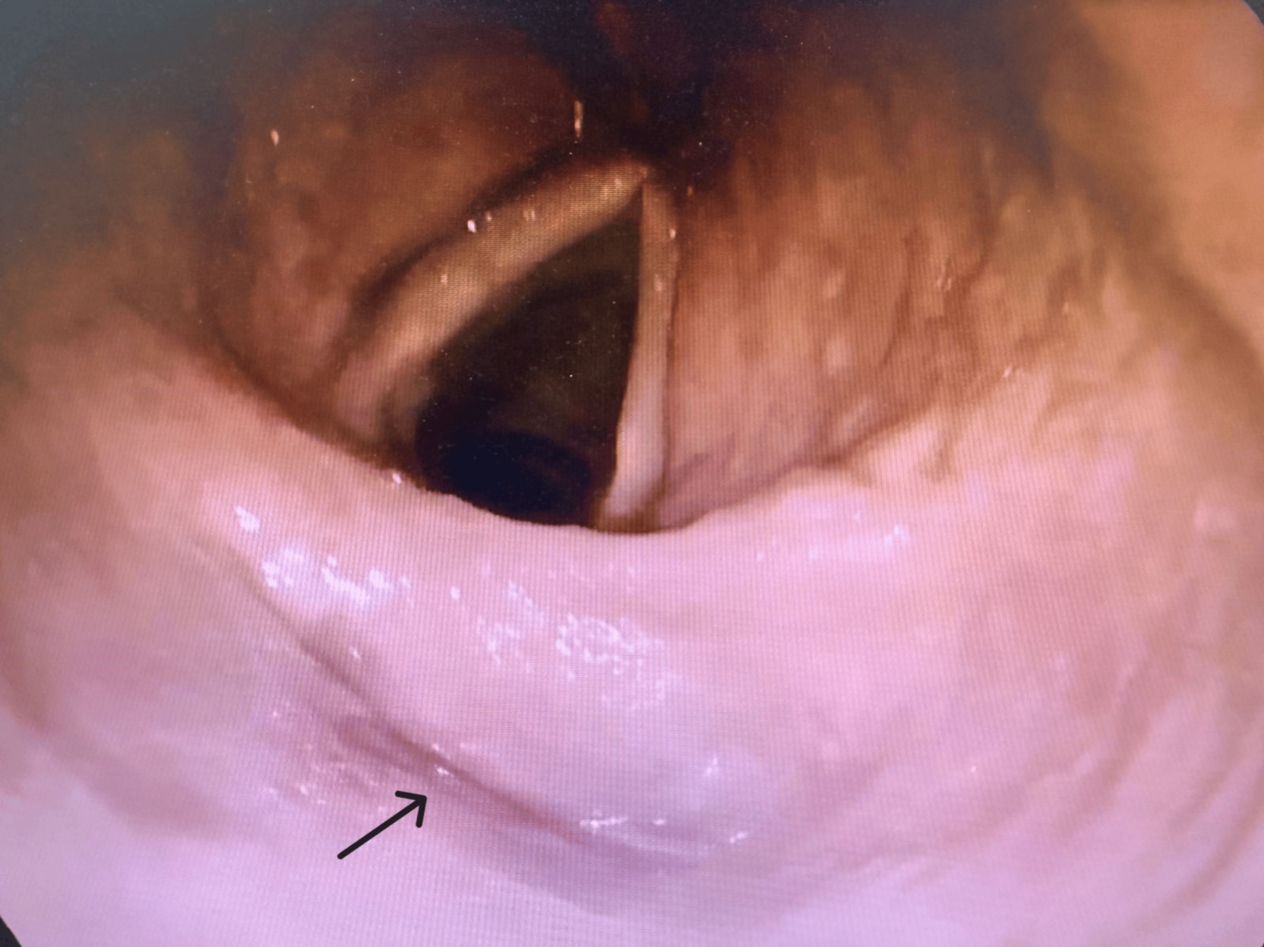
Gastroscopy showing collapsed esophageal opening (arrow)

**Figure 2:**
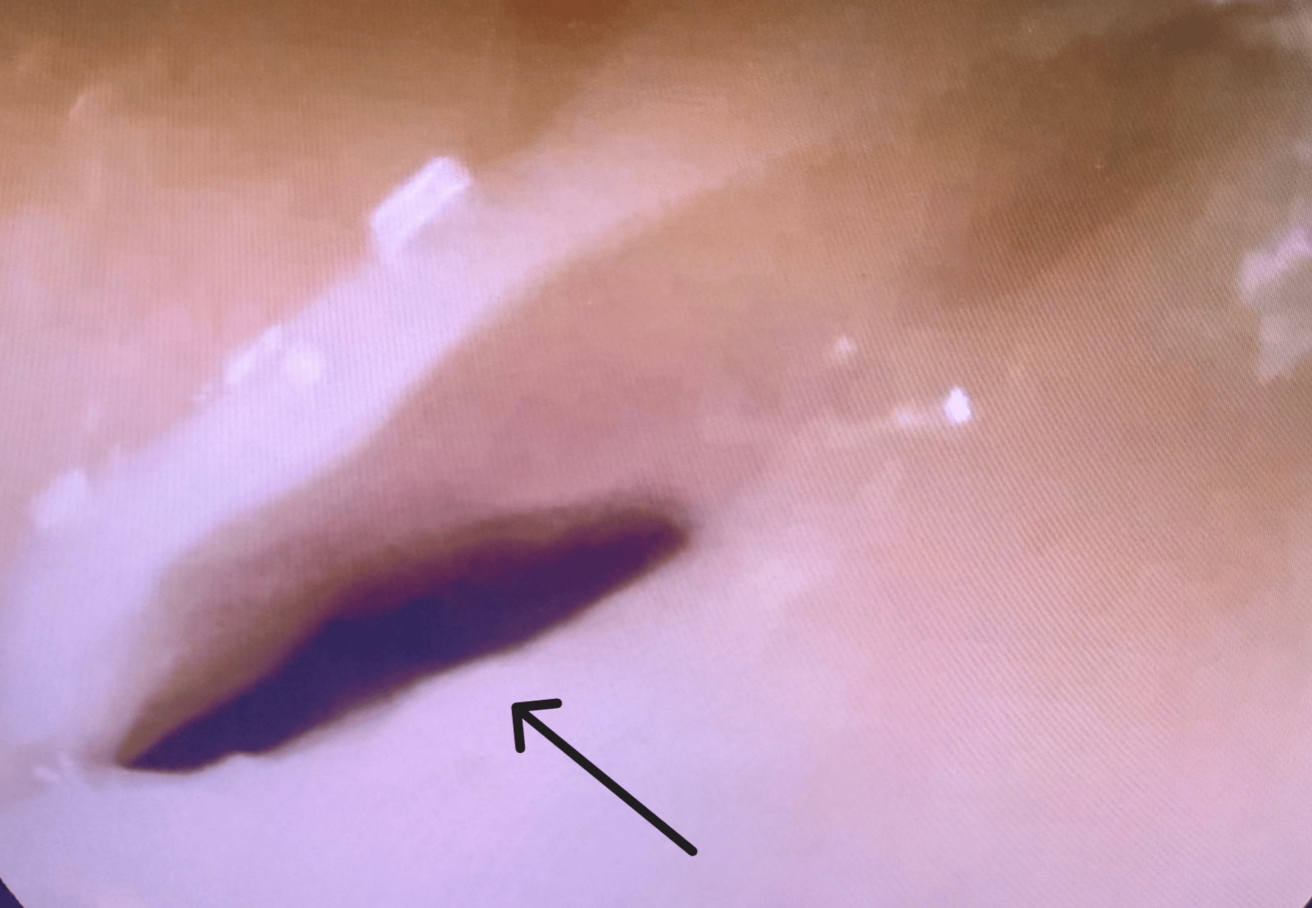
Severe narrowing of the esophageal lumen preventing passage of the scope (arrow)

Gastroenterology was consulted for the worsening dysphagia, which had progressed to complete intolerance of oral intake. Attempts to pass a nasogastric tube were unsuccessful, as it could not advance beyond the upper esophageal sphincter. Esophagogastroduodenoscopy revealed severe narrowing at the level of the upper esophageal sphincter, preventing further passage of the scope; no intraluminal masses or malignancies were observed (Figures 1, 2).

**Figure 3:**
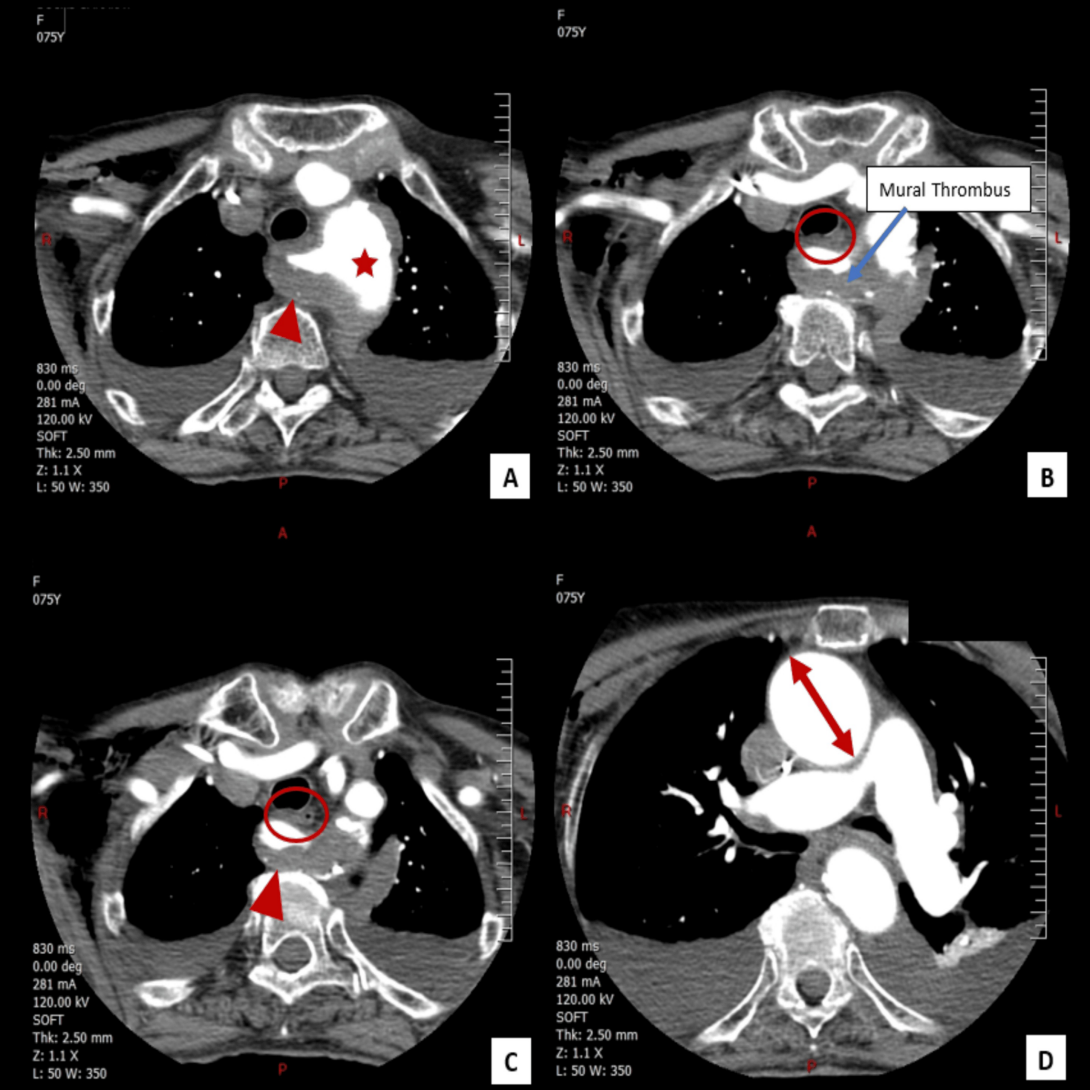
Multiple CT scan cuts of the neck and chest with IV contrast showing an aberrant right subclavian artery This axial CT scan illustrates a case of dysphagia lusoria with an aberrant right subclavian artery (arrowhead) originating from the right side of the aortic arch (star) (Figure A) and coursing posteriorly and laterally, compressing the esophagus (circle) (figures B and C). The anomalous course of the subclavian artery is seen causing an external indentation of the esophageal lumen, which is a classic feature of dysphagia lusoria. Additionally, there is a 2-cm thoracic aneurysmal dilatation of the aortic arch (double-headed arrow) (figure D) and the proximal segment of the right subclavian artery, with associated peripheral non-occlusive mural thrombus extending into the right subclavian artery (figure B). The thrombus is seen adhering to the inner wall of the artery but does not obstruct the lumen.

Given the failure of these interventions and the impossibility of performing a barium swallow imaging due to the complete intolerance of oral intake, even liquids, advanced imaging was pursued. A contrast-enhanced computed tomography (CT) scan of the neck and chest revealed an ARSA originating from the aortic arch, following a retroesophageal course and compressing the esophagus posteriorly. The artery was significantly enlarged, with a maximal diameter of approximately 2 cm, and demonstrated marked parietal thrombosis. These findings confirmed the diagnosis of dysphagia lusoria, with esophageal compression resulting from the thrombosed and dilated aberrant artery (Figure 3).

Upon this diagnosis, a vascular surgeon was promptly consulted to discuss potential management options. The surgeon recommended surgical correction; however, due to the patient's critically ill state in the intensive care unit, she was not considered a candidate for surgical intervention. Despite medical management and intensive care, the patient developed septic shock and unfortunately passed away.

## Discussion

Dysphagia lusoria is a rare condition characterized by esophageal compression due to an ARSA [7]. This vascular anomaly occurs in approximately 0.5-1% of the population and is often asymptomatic. When symptoms do occur, dysphagia is the most common presentation, typically resulting from mechanical obstruction [8]. The term “dysphagia lusoria” was first introduced by David Bayford in the 18th century to describe dysphagia caused by an ARSA [9]. While many cases are mild and managed conservatively, complications such as aneurysmal dilation or thrombosis can lead to severe symptoms, including complete esophageal obstruction [10]. In this patient, thrombosis of the ARSA led to significant esophageal compression, resulting in complete obstruction.

Imaging is crucial for diagnosing dysphagia lusoria. CT angiography and magnetic resonance angiography are particularly useful for evaluating the retroesophageal course of the aberrant artery and identifying complications such as thrombosis or aneurysmal dilation [11]. In this case, CT imaging demonstrated the aberrant artery and its associated thrombosis, providing a clear explanation for the patient’s symptoms and their progression.

Management of dysphagia lusoria depends on symptom severity. Many asymptomatic or mild cases are managed conservatively with dietary modifications and monitoring. However, in severe cases with significant symptoms or complications such as aneurysm or thrombosis, surgical intervention is often necessary [11]. Surgical options include resection or bypass of the aberrant artery to relieve esophageal compression and prevent further complications [12]. In this patient, conservative measures were unsuccessful due to the severity of the obstruction, prompting referral for vascular surgical evaluation.

A review of similar cases indicates that thrombosis of an ARSA is a rare but documented complication [13]. Previous reports describe cases of ARSA thrombosis causing severe dysphagia and associated weight loss, as well as successful surgical management resulting in symptom resolution [14]. For instance, Bellamkonda et al. reported a case where a patient with ARSA thrombosis experienced significant dysphagia and weight loss, which improved after surgical intervention [15]. Similarly, Cobos-González et al. documented a case of dysphagia lusoria caused by ARSA, where the patient benefited from hybrid surgical management [16].

This case underscores the importance of early recognition and appropriate diagnostic imaging in the evaluation of unexplained dysphagia. ARSA, although rare, should be considered in the differential diagnosis, particularly in older adults with progressive dysphagia. Timely identification of such vascular anomalies can facilitate appropriate management strategies and potentially prevent complications associated with delayed diagnosis and treatment.

## Conclusions

This case highlights a rare instance of dysphagia lusoria, which was further complicated by the rare occurrence of thrombosis of an ARSA, resulting in complete esophageal obstruction. While the condition is typically mild, complications such as thrombosis or aneurysm can lead to life-threatening presentations requiring advanced imaging and multidisciplinary care. Early detection of the cause of dysphagia by simple radiological procedures, such as barium swallow, is crucial. Clinicians should maintain a high level of clinical suspicion for vascular anomalies that can result in esophageal compression. Early recognition of this condition in patients with unexplained dysphagia and weight loss, especially in the elderly, is critical for initiating appropriate management. Definitive treatment often requires vascular surgical intervention, as demonstrated in this case.
